# The efficacy of commercially available essential oil-based candles as spatial repellents against *Aedes aegypti* mosquitoes – do they actually repel?

**DOI:** 10.3389/finsc.2026.1917728

**Published:** 2026-08-26

**Authors:** April D. Lopez, Claudia J. Galvan, F. Omar Holguin, Christopher J. Sroka, Immo A. Hansen

**Affiliations:** 1 Department of Biology, New Mexico State University, Las Cruces, NM, United States; 2 Department of Plant and Environmental Sciences, New Mexico State University, Las Cruces, NM, United States; 3 Department of Economics, Applied Statistics, and International Business, New Mexico State University, Las Cruces, NM, United States

**Keywords:** *Aedes aegypti*, candle, essential oil, mosquito, spatial repellent, taxis cage

## Abstract

Spatial mosquito repellents contain volatile compounds that disrupt mosquito host seeking behavior. Some essential oils have been shown to be effective spatial repellents against biting insects. In this study, we acquired seven batches of commercially available candles containing mixtures of essential oils. Six of these candle labels claim that they repel mosquitoes. We used a taxis cage assay to evaluate the spatial repellency against *Aedes aegypti* mosquitoes of these and two control candles without essential oils. We used Headspace Solid-phase Microextraction Gas Chromotography-Mass Spectrometry analysis to identify a putative list of volatile organic compounds emitted by heated candle wax. We subsequently correlated individual compounds with spatial repellency. Three types of candles, containing mixtures of various essential oils, showed strong repellent efficacy compared to the controls, while the other three types did not produce significant repellency. The volatile organic compound composition varied between the different types of candles. Candles that were efficient mosquito repellents contained relatively high concentrations of essential oils. We identified several volatile organic compounds that are positively associated with mosquito repellency. The results of this study show that the efficacy of essential oil-based candles as mosquito repellents is based on their formulation.

## 1 Introduction

The burden that mosquito-borne diseases pose on the global population continues to rise (1). One way to protect people from mosquito bites is by the use of mosquito repellents. Topical and spatial repellents are products that contain compounds that modify mosquito behavior, thereby providing personal protection against mosquito bites (2).

Mosquito repellents interfere with the host-seeking of mosquitoes in two main ways - either by masking host odors or by activating mosquito chemoreceptors to repel mosquitoes (3). They can be divided in two types – contact repellents and spatial repellents. Contact repellents are applied to a host’s skin or clothing. The WHO classifies spatial repellents as products that disperse volatile compounds into the air and can be placed in or around houses to disrupt mosquito host-seeking behavior (4). Currently, all WHO recognized spatial repellent products are pyrethroid-based (1, 4, 5). Pyrethroids are compounds commonly used as insecticides. MosquitoShield™ (transfluthrin) (5), OFF!^®^ Mosquito lamp (metofluthrin) (6), OFF!^®^ Clip-On (metofluthrin) (7), and ThermaCELL^®^ (allethrin) (8) have shown spatial repellent efficacy in field studies. However, adverse effects from prolonged exposure have been observed in rodents which causes concerns for human health (9–12).

Mosquito repellent candles are commonly used as mosquito repellents in the US (13). There is a growing demand for ‘natural’ or plant-based repellent products. Concurrently, a plethora of essential oil-containing candles with label claims of mosquito repellency are readily available for consumers. Plant essential oils are a complex mixture of organic compounds (14). Compound composition varies depending on the type of plant used for extraction, the plant part used, the geographical location, and seasonality of the harvest (15).

Several studies have investigated the repellency of citronella, geraniol, cinnamon, rosemary, and clove oil as mosquito repellents as both topical and spatial repellents (16–19). However, there are relatively few studies have evaluated the efficacy of essential oil candles as spatial repellents. Müller et al. (20) formulated essential oil candles and used human landing assays to determine the efficacy of geraniol, linalool, and citronella candles in high biting pressure environments against mosquitoes and sandflies. They demonstrated geraniol and linalool candle formulations as efficacious at reducing mosquito landings. In a separate study, Müller et al. (21) evaluated geraniol, linalool, and citronella essential oil as spatial repellents in both diffuser and candle formulations and found that geraniol was an effective spatial repellent in both diffuser and candle devices. In a third study Müller et al. (20) evaluated 5% geraniol, linalool, and citronella oil candles as repellents against mosquitoes and sandflies in a field setting using both Centers for Disease Control and prevention US Public Health Agency (CDC) traps and human landing assays. They placed candles at either 1, 2, or 3 meters away from CDC traps and observed significant increases in the number of biting insects caught at farther distances for all three candles. In the volunteer landing assay, the geraniol candle significantly decreased the biting pressure in both high- and low-pressure environments. These studies evaluated laboratory formulated candles containing specific concentrations of individual essential oils. There are few comparative studies that test commercially available essential oil candles as spatial repellents against mosquitoes. Lindsay et. al., conducted a field study to evaluate a commercially available candle (3% citronella essential oil) as a repellent against various *Aedes* spp. mosquitoes (22). In field tests, volunteers were placed in between two citronella candles and asked to count the number of mosquito bites received. Volunteers reported a 42% decrease in mosquito bites using the citronella candle compared to a control candle without essential oils. In contrast, Revay et. al., evaluated commercially available Cutter^®^ Crito Guard 3% citronella candle in a field setting, and did not observe repellency of *Aedes, Culex, or Anopheles* genera mosquitoes compared to an untreated control (23). Similarly, Rodriguez et. al., reported no repellency of *Aedes aegypti* mosquitoes with a Cutter^®^ Crito Guard 3% citronella candle using a taxis cage assay placed inside a wind tunnel (24).

The present study evaluates the efficacy of seven commercially available essential oil-containing candles as spatial repellents against *Ae. aegypti* mosquitoes. We sourced commercially available candles that are marketed as mosquito repellents and used a taxis-cage assay to measure the percent attraction of mosquitoes to a human volunteer in the presence or absence of a candle.

## 2 Materials and methods

### 2.1 Mosquito rearing

*Ae. aegypti* UGAL (University of Georgia Laboratory) strain mosquitoes were used for this study. The UGAL strain was gifted from Alexander Raikhel’s laboratory at the University of California Riverside. Approximately 600 eggs were hatched in 32 × 42 × 6 cm^3^ pans filled with 2.5L of deionized water. Cat food pellets (Special Kitty, Walmart Stores Inc., Bentonville, AR, USA) were fed to larvae ad libitum and the water was changed as needed. The larvae were kept in an incubator with a 14/10 hour light/dark cycle respectively and set to 27.8 °C and 74% relative humidity. Pupae were transferred into a 200mL dish filled with deionized water and placed in a 30 × 30 × 30 cm^3^ BugDorm-1 Insect-Rearing cage (Bug Dorm Company, Taichung, Taiwan) and allowed to emerge. Adults were kept in an insectary with 14/10-hour light/dark cycle respectively and set to 27.8 °C and 74% relative humidity and unlimited access to 20% sucrose solution in a 25mL Erlenmeyer flask with a cotton wick.

### 2.2 Candle acquisition

All candles were acquired through Amazon.com (USA) (**Table 1**). The details of each product label can be found in **Table 1** including: the candle name used for this study, the trade name and brand name, wax base, essential oils in the candle, and the total essential oil percentage in each candle. Not all ingredient information was available, in which case was labeled as unknown. Images of the candles and pertinent packaging information are presented in **Supplementary Table 1**.

**Table 1:** Product information for all candles tested.

Candle name	Trade name and brand name	Wax base	Essential oils	Percentage of total essential oil in the candle
Soy	Emergency Candle Red-E Home	Unscented soy wax	None	None
Bee’s Wax	Hope & Need Unscented Candle	Bee’s wax	None	None
EO1	Mosquito Naturals Fresh Mint	Soy	Peppermint, Wintergreen, Citronella, Lavender, and Rosemary	Unknown
EO2	Mosquito Naturals Lemongrass	Soy	Lemongrass, Citronella, Geranium, Lemon	Unknown
EO3	OFF!^®^ Citronella Candle	Soy and Paraffin	Citronella	3% total
EO4	Murphy’s Naturals^®^ Mosquito Repellent Candle	Soy and Bee’s wax	Rosemary, Peppermint, Citronella, Lemongrass, Cedarwood	5% total
EO5	Fabulous Frannie Essential Oil Candle Bug Away	Soy	Citronella, Lavender, Eucalyptus, Lemongrass.	Unknown
EO6	Cutter^®^ Outdoor Candle	Soy and Paraffin	Citronella, Corn mint	4.5% total
EO7	Bug Bane^®^ Mosquito Repellent Candle	Soy and Bee’s wax	Rosemary, Peppermint, Citronella, Lemongrass, Cedarwood	5% total

### 2.3 Taxis cage assay

**Figure 1:**
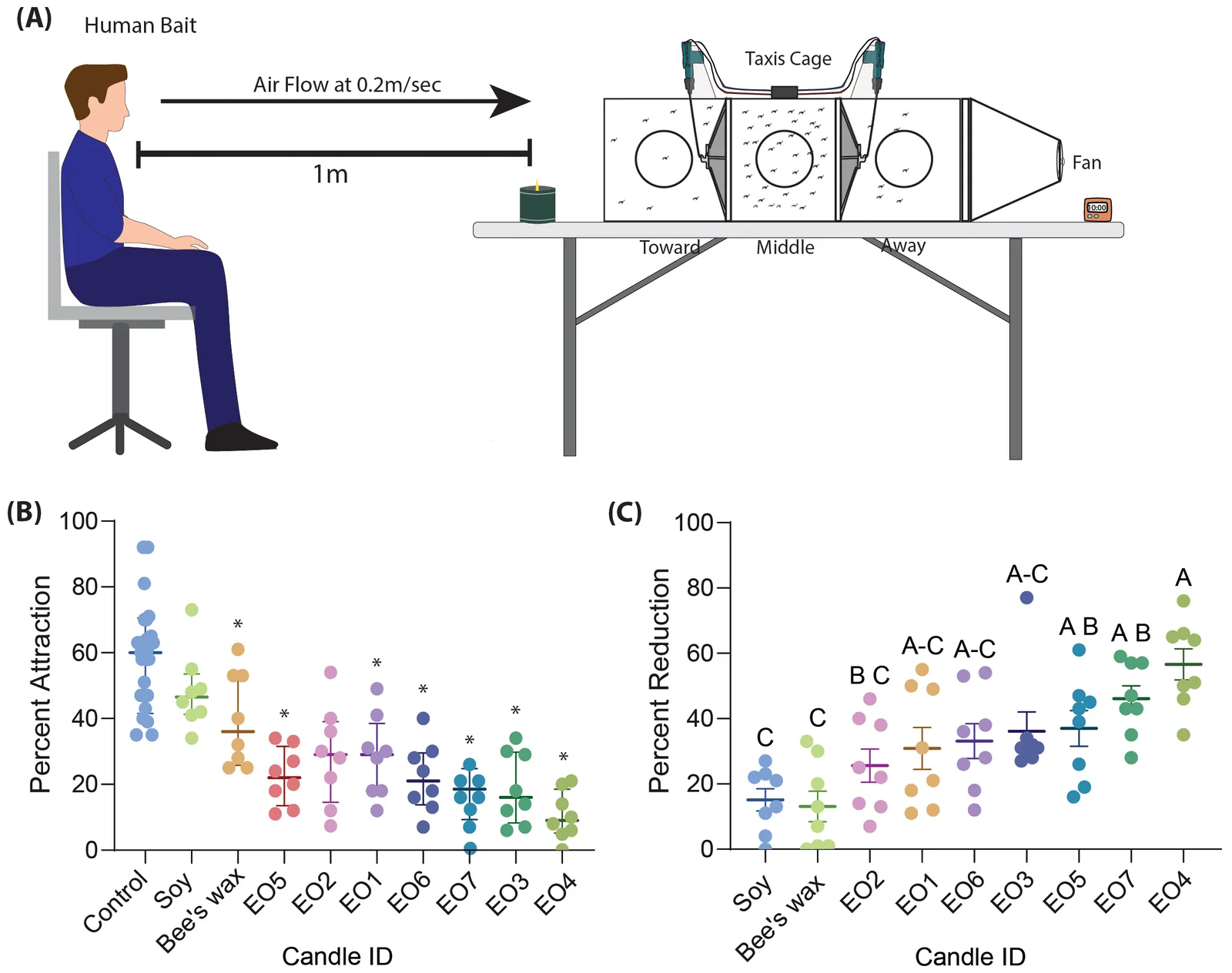
Essential oil containing candles reduce attraction of *Ae. aegypti* mosquitoes to a human bait. **(A)** Taxis cage assay set-up. Source: adapted from Luker (25), licensed under CC BY 4.0. **(B)** Shown is the percent attraction of mosquitoes to a human volunteer (n=8). Individual data points are overlaid with median and interquartile range bars. Paired t-tests were used to compare the mean percent attraction between each candle to its corresponding control. Asterisks indicate statistical significance such that p<0.05. **(C)** Shown is the percent reduction in attraction of mosquitoes to a human volunteer in the absence and presence of essential oil-containing candles (n=8). The data was analyzed as described above. A one-way ANOVA followed by a Tukey’s multiple comparison test was used to analyze significance between the means of each group to one another. Groups that share letter ranges show no statistical difference and groups that differ in all letters are significantly different from one another.

A 90×31×31cm^3^ three-chamber custom-built taxis cage assay was used to measure spatial repellency (**Supplementary Figure 1**). **Figure 1A** was adapted from Luker (25) and edited in Adobe Illustrator CC 2025 [software] (Adobe Inc., San Jose, California, USA). The taxis-cage was split into three interconnected chambers by 5×5cm^2^ remote-operated Plexi-glass doors. A 31×21×21cm^3^ funnel was attached to the ‘away’ side of the taxis-cage with a 12×12×2cm^3^ 12V computer fan (Wathai, Amazon, USA) that propelled wind though the taxis cage toward the ‘away’ side. The wind speed was kept constant at 0.2 m/s, measured using a digital hot-wire anemometer (The Value Leader, Beaverton, OR, USA). All trials were conducted in an indoor laboratory setting. The taxis-cage set up was placed on a 8ft plastic folding table. *Ae. aegypti* UGAL strain mosquitoes 10–14 days old were sugar-starved for at least 4 hours prior to experimentation. An ambient temperature of 22-27°C and 40% humidity was maintained throughout experimentation.

Candles were lit 30 minutes prior to experimentation. 50 female mosquitoes were aspirated into a collection vial and immediately placed into the middle chamber while chamber doors remained closed and allowed to acclimate for 2 minutes. A volunteer was asked to remove shoes and sit 1m upwind from the taxis cage. A pre-lit candle was placed on the Table 31cm away from the taxis-cage and between the taxis-cage and the volunteer immediately before the experiment. The chamber doors were opened via remote control, and mosquitoes were allowed to roam freely for 10 minutes. At the end of the experiment, the chamber doors were closed and mosquitoes from each chamber were collected and counted. Before each candle test, a control test, without a candle, was used to assess the overall attraction the mosquitoes had to the particular volunteer. If a minimum threshold of 34% attraction was not achieved, then the experiment was rescheduled for a different day. For each candle, 7 trials were conducted with at least 3 male and 3 female volunteers per candle.

### 2.4 Taxis-cage assay statistics

Percent attraction was calculated as the number of mosquitoes in the ‘toward’ chamber divided by the total number of mosquitoes used in the experiment, multiplied by 100.

(1) $$Percent\;Attraction\;(PA)=\frac{N_{\left(Towards\right)}}{N_{\left(Total\right)}}\;\times 100$$

The percent reduction in attraction was calculated by subtracting the percent attraction of the control trial and the percent attraction of the candle trial per individual volunteer.

(2) $$Percent\;Reduction\;=PA_{control}-PA_{Candle}$$

All statistical analyses were conducted in GraphPad Prism 10.0.0 (26). Percent attraction was measured for each test subject with both a candle absence and candle presence; therefore, paired t-tests were used to compare the percent attraction of mosquitoes to a test subject between the control and candle trial for each candle type. To adjust for the variation in mosquito attraction to different test subjects, we calculated percent attraction. A One-way Analysis of Variance (ANOVA) followed by Tukey’s *post hoc* test (α=0.05) was used to compare the reduction in attraction between each candle type. Statistical significance was displayed using the compact letter function such that candle types that share a letter are not statistically different from one another while those that do not share a letter are statistically significantly different from one another.

### 2.5 Ethics declaration

All experiments conducted in this study have been reviewed and approved by the New Mexico State University Institutional Review Board (IRB). We confirm that we followed all guidelines mentioned in our current application— (22010) “Insect and Tick-Repellent Research”, which expires 09/2029. Vulnerable persons (i.e., minors, pregnant and nursing women, prisoners, and immune-compromised individuals were excluded from this study. Participants were advised to avoid alcohol, tobacco, and any scented products at least 12 h prior to this study. Seven volunteers with a balanced ratio of males and females (3:4) were recruited for testing. Volunteers were between 21 and 54 years old.

### 2.6 GC-MS analysis

#### 2.6.1 Sample preparation

Wax samples were prepared by shaving off 100mg of unburned candle into a round bottom 20mL glass headspace vial with 18mm magnetic screw caps and silicone septa (Shimadzu Scientific Instruments, U.S. Webstore). Volatile Organic compound (VOC) extraction was fully automated using an AOC-6000 Plus PAL3 Autosampler system configured for high-throughput sample processing. Samples contained in 20mL headspace vials were transferred to the automated incubator/agitator module. Prior to extraction, the samples were equilibrated and preconditioned at 40 °C for 5 minutes under continuous thermal mixing at 250 rpm. Following the incubation period, headspace extraction was executed using a Restek PAL Smart SPME Arrow Tool equipped with a 1.10mm PDMS Smart SPME Arrow fiber (Phase thickness 100um, Phase Length 20 mm (Part No 28906-3). The SPME Arrow penetrated the sample vial septum at a controlled speed of 20 mm/s to a final target depth of 33mm. The Arrow fiber was exposed to the sample headspace for 10-minute extraction under sustained thermal and agitation conditions to isolate the target analytes.

#### 2.6.2 Injection and thermal desorption

Following the extraction cycle, the tool retracted and transferred the analytes directly to the Gas Chromatography (GC) injection port at a penetration speed of 100mm/s to an injector depth of 54mm. Thermal desorption of the sorbent phase was performed with a 2-minute desorption period. Pre- and post-conditioning sequences were fully automated to ensure rigorous thermal cleaning cycles of the Arrow fiber between sequential sample runs.

#### 2.6.3 HS SPME GC-MS instrumental analysis

Chromatographic separation and mass spectral detection were performed on a Shimadzu Gas Chromatography Mass Spectrometry (GC-MS)-TQ8050 system coupled with a triple quadrupole mass spectrometer system operated in electron ionization (EI) mode. Separation was achieved on a Shimadzu DB-5sil MS capillary column (30m x 0.25mm i.d., 0.25 μm film thickness). The GC oven temperature program was initiated at 60 °C and increased at 3 °C/minutes to 246 °C (held for 15 min) until the end of the analysis. Helium was used as the carrier gas. The split injector temperature was maintained at 250 °C. The transfer line was maintained at 280 °C, and the ion source at 200 °C. Ionization was generated with an electron kinetic energy of 70eV. Mass spectra and reconstructed total ion chromatograms (TICs) were acquired via automated scanning across a mass range of m/z 41.00-415. All GC-MS data were processed with MS-Dial 5.5.251021 (**Supplementary File 2**) (27). Compound identifications were putatively assigned by spectral matching to the Adams Essential Oil GC-MS library version 4 (Diablo Analytical, Inc) using total spectrum score of greater than or equal to 0.9and retention index (RI) tolerance of less than or equal to 10 (**Supplementary File 3**). Representative chromatograms from each sample can be found in **Supplementary Figure 2**.

#### 2.6.4 Metabolomic statistical analyses

**Figure 2:**
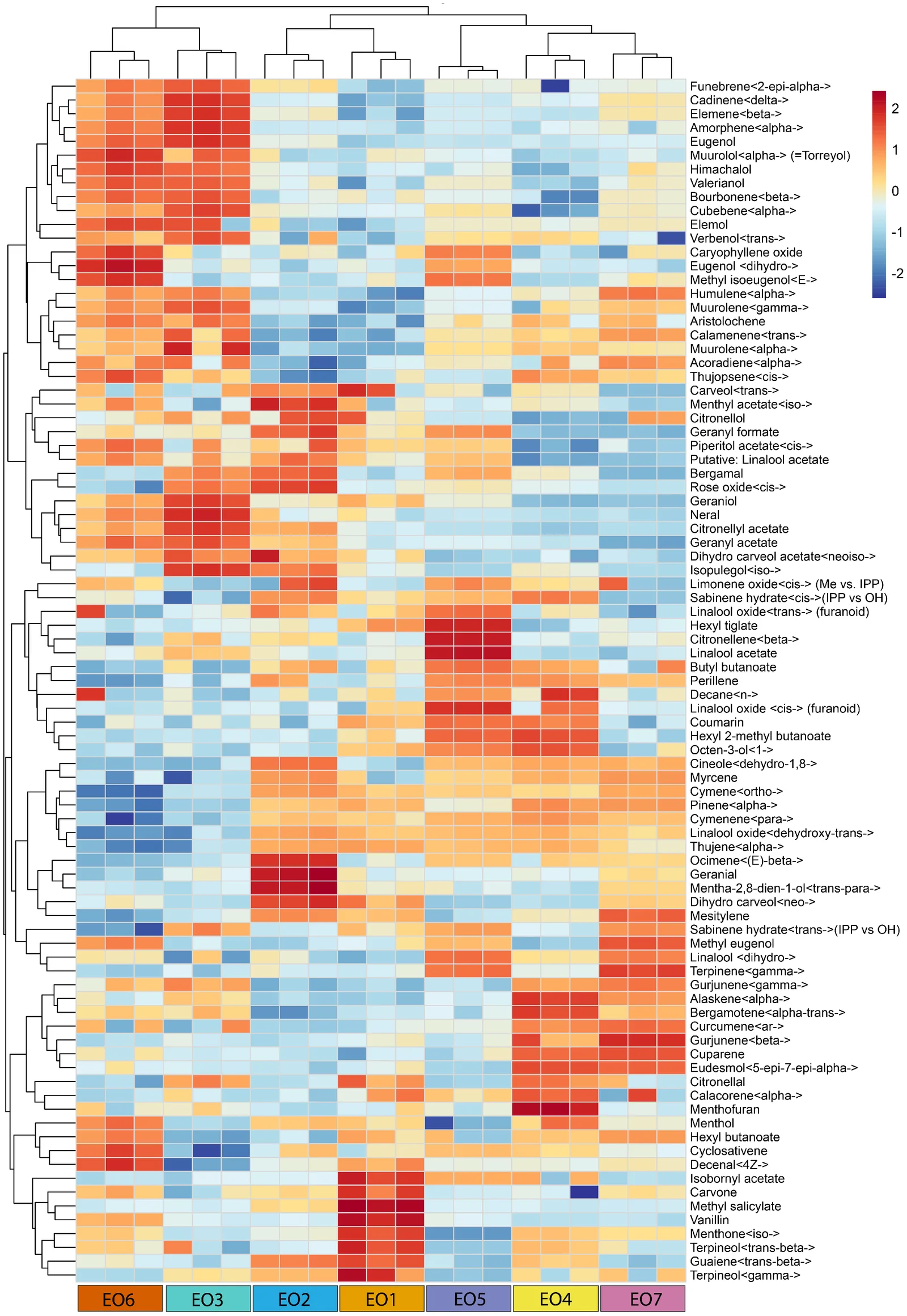
High variability in the relative abundance of compounds in different essential oil-containing candles. Heatmap of identified compounds in each candle. The relative abundances are normalized to the sum of each replicate, log10 transformed, and scaled. Cells in blue represent relatively low percent peak area while cells in red represent high percent peak area in each sample. The colored bars at the bottom of the graph mark the three replicates of each essential oil-containing candle analyzed.

**Figure 3:**
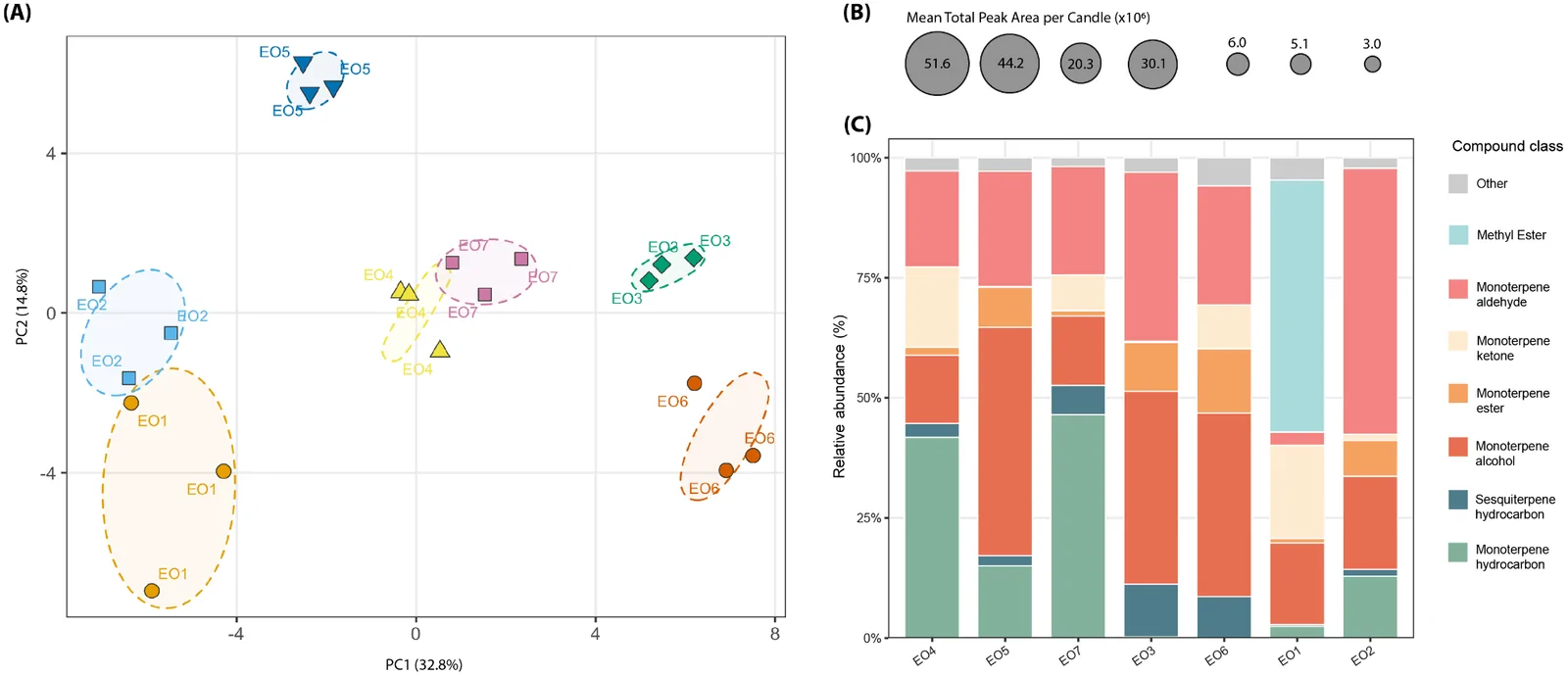
The essential oil containing candles used in this study differ in metabolic profiles. Shown are the results of a Head-Space GC/MS Compound Profiling of Candles. **(A)** Principal component analysis of candle compound profiles. **(B)** The mean total peak area per candle is reported inside or above the circles, relative to the size of the circle. **(C)** The relative distribution of compound classes in each candle.

The following statistical analyses were conducted in RStudio.4.5–2 software (Posit team. Boston, MA). Prior to statistical analysis, the data was transformed by log10, normalized to sum, and mean-centered and divided by the standard deviation of each variable. The data in **Supplementary File 2** was used to construct the heatmap with the R package *pheatmap* and the PCA analysis was conducted with the *BaseR* package (**Figures 2**, **3A, B**). The data found in **Supplementary File 3** was used to construct **Figure 3C** using the *BaseR* package. Because of the large number of compounds identified from the Headspace Solid-phase Microextraction Gas Chromotography-Mass Spectrometry (HS SPME GC-MS) analysis relative to the number of human subjects, LASSO was used to identify a small set of compounds that are highly predictive of repellency. LASSO was conducted in R using the *glmnet* package. Ten-fold cross-validation was used to determine the LASSO shrinkage penalty that minimizes the mean squared error. The final LASSO model was fit on the entire dataset using that shrinkage penalty. All final figures were prepared using the Adobe Illustrator software package mentioned above.

## 3 Results

### 3.1 Spatial repellency of essential oil candles

We used a taxis cage assay (**Figure 1A**) to measure the percent attraction of mosquitoes to a human volunteer (bait), which was calculated by **Equation 1** (see Methods section). In the absence of a candle, an average of 60% of mosquitoes were attracted to a human bait across all tests (**Figure 1B**). All candles, except Soy and EO2, significantly reduced attraction compared to the non-candle control (Paired t-tests, p<0.05). Reduction in attraction (**Figure 1C**), was calculated using **Equation 2** (see Methods section). We observed less than 15% reduction in attraction for both soy and bee’s wax controls. EO4, EO5, and EO7 had a mean 56%, 40%, and 45% reduction in attraction, respectively, which was significantly higher than both bee’s wax and soy controls. (One-Way ANOVA; F = 6.29, p<0.05).

To normalize the percentage attraction for each volunteer, we calculated percent reduction in

### 3.2 Compound profiling of essential oil-containing candles

Since essential oils are complex mixtures of various plant secondary metabolites, we used HS SPME GC-MS of candle wax to putatively identify each component based on retention index and spectral matching to the Adams Essential Oil spectral library (**Supplementary File 2**). A total of 87 compounds were putatively identified across seven essential oil candles. The heatmap in **Figure 2** shows the relative abundance of each compound per replicate of each candle. To assess the variation in metabolic profiles of all the candles relative to one another, we performed a PCA analysis. We observed separation between each candle and clustering of replicates for each candle (**Figure 3A**). We then calculated the mean total peak area per candle to quantify the relative number of compounds present in each sample (**Figure 3B**). High repellency candles had higher mean peak compared to intermediate and low repellency candles. We categorized each compound into chemical classes (**Figure 3C**; **Supplementary File 3**) and observed a relatively high proportion of monoterpenes across all candles. EO4 and EO7 had relatively higher proportion of monoterpene hydrocarbons compared to the other candles.

### 3.3 LASSO prediction of repellent compounds

The LASSO model identified five compounds that are most predictive of mosquito repellency. These compounds and their estimated coefficients are shown in **Table 2**. Citronellal, trans-α-bergamonte, and curcumene were positively associated with repellency, as their coefficient estimates were positive values, while dihydro carveol acetate and piperitol acetate were negatively associated with repellency.

**Table 2:** Compounds selected by the LASSO model as the most predictive of repellency and their estimated coefficients.

Compound	Coefficient
Citronellal	7.2878465
Bergamotene<alpha-trans->	7.0343126
Curcumene<ar->	0.9561576
Dihydro carveol acetate<neoiso->	-1.7781545
Piperitol acetate<cis->	-1.3232564

## 4 Discussion

Smoke from burning plants has been used as a spatial repellent against insects for centuries in the form of smudge fires or burning incense (17, 28–30). Essential oil-containing spatial repellents have been around for centuries. The first citronella candle, marketed to repel mosquitoes and other insects, was patented in 1943 (patent number: US2323804A). Modern mosquito repellent candles typically contain mixtures of several essential oils as do the candles we used in this study (see **Table 1**). Two main considerations limit the type and the concentration of essential oils in commercially-produced candles – the high cost of some essential oils, and the physiochemical limits in the essential oil holding capacity of wax.

We designed the taxis cage assay we used in this study in order to maximize the measurable effect of spatial repellents while minimizing confounding variables (24). This assay is extremely sensitive in detecting repellency. We observed that even a bee’s wax candle slightly reduced mosquito attraction to a volunteer. While this assay is designed to optimize the repellent effects of spatial repellents, it has to be mentioned that it excludes several environmental variables that are relevant under outdoor conditions such as temperature, humidity, mosquito abundance, biting pressure, wind speed, wind direction, optical cues, and human movement.

As mentioned above, earlier studies of essential oil-containing candles have resulted in ambiguous results (3, 20, 21, 23, 24). Our findings clearly show that certain essential oils contribute to the repellent efficacy of candles against *Ae. aegypti* mosquitoes.

For our headspace HS SPME GC-MS analysis, we included candles that reduced mosquito attraction greater than twenty percent in the taxis cage assay. Although several candles contained the same essential oils, we observed substantial differences in the compound abundance across different candle types. PCA analysis demonstrated a clear segregation of EO4, EO5, and EO7 candles from the remaining candles, indicating that these high-repellent candles are compositionally distinct from the others (**Figure 3**). It’s important to note that EO4 and EO7 list identical active ingredients, yet their chemical profile doesn’t completely overlap. This could be in part because the source and concentration of essential oils along with variations in the manufacturing processes can affect the compound compositions. In general, we found that high repellent candles had greater mean peak areas per candle, suggesting that high concentration of active ingredients may contribute to repellency. Notably, candle composition including active ingredient concentrations, wax type, and wick type may affect the volatile compound emission rate (31).

We used a LASSO model to putatively identify compounds that are the strongest predictors of spatial repellency while screening out compounds that may have a weak or no relationship with repellency while accounting for the high dimensional nature of our HS SPME GC-MS data. The identification of two compounds that are strong predictors with negative coefficients suggests that some compounds or combinations of compounds may reduce overall repellency outcomes. These relationships can be experimentally tested. It is worth noting that other compounds beyond those listed in **Table 2** may still contribute to repellency.

The LASSO model predicted citronellal, trans-α-bergamontene, and ar-curcumene as the strongest predictors of repellency. Citronellal was present in high abundance in EO4, the candle that offered the highest repellency. Both ar-curcumene and trans-α-bergamontene were present in high abundance in both EO4 and EO7, the two candles that offered the greatest reduction in attraction. Citronellal is a monoterpene aldehyde and a major constituent of essential oils derived from several *Cymbopogon* spp. plants such as citronella and lemongrass which are known for their mosquito repellent properties. Several studies have demonstrated repellent efficacy of Citronellal against various mosquito species (32–34). Trans-α- bergamotene is a sequisterpene, and a constituent of several essential oils from plants such as cedarwood and lavender that are known repellents (35, 36). Studies investigating bergamotene isoforms as mosquito repellents are lacking. Ar-curcumene is a sesquiterpene present in cedarwood, citronella, and peppermint essential oils, which have all been demonstrated as mosquito repellents. Studies investigating the repellency of ar-curcumene are lacking; however, one study reports mosquito larvicidal effects (37).

Overall, our findings demonstrate that essential oil candles can effectively reduce mosquito attraction. Further investigation of individual compounds and defined compound mixtures are needed to identify all active ingredients responsible for repellency. Also, while the taxis cage test is great to measure candle repellency in the laboratory, field testing of repellent candles under different environmental conditions is advisable to inform label claims of repellency.

## Data Availability

The original contributions presented in the study are included in the article/**Supplementary Material**. Further inquiries can be directed to the corresponding author/s.
