# Supplementary material for: The efficacy of commercially available essential oil-based candles as spatial repellents against Aedes aegypti mosquitoes – do they actually repel?
Source: Front Insect Sci. 2026 Aug 26;6:1917728. doi: 10.3389/finsc.2026.1917728 (PMC13555344; doi:10.3389/finsc.2026.1917728)
Supplement: Supplementary file 1 [file SupplementaryFile1.docx]

**Supplemental Figure 1: Taxis-Cage Setup.**

 **Supplemental Figure 2: Representative stacked GC-MS total ion chromatograms (TICs) of the five essential oil candles (EO1–EO5).** Peak labels correspond to the major putatively identified volatile essential oil constituents and match the compound numbers reported in Supplemental Table 2. The annotated peaks and their corresponding experimental retention indices (RIs) are as follows: 2 (RI = 933.52), 7 (RI = 991.17), 26 (RI = 1147.41 ), 34 (RI = 1191.39), 40 (RI = 1227.55), 42 (RI: 1249.56) and 45 (RI: 1266.34). A complete list of all identified chromatographic components, including compound identities, chemical classifications, experimental and library retention indices, and mass spectral identification parameters used for compound identification, is provided in Supplemental Table 2. Relative abundances can be found in Supplemental File 2.
