# Supplementary material for: The efficacy of commercially available essential oil-based candles as spatial repellents against Aedes aegypti mosquitoes – do they actually repel?
Source: Front Insect Sci. 2026 Aug 26;6:1917728. doi: 10.3389/finsc.2026.1917728 (PMC13555344; doi:10.3389/finsc.2026.1917728)
Supplement: Supplementary file 2 [file Table1.docx]

**Supplemental Table 1: Images of candle types.**

| Candle | Pertinent Packaging | Labeled Active  Ingredients |
| --- | --- | --- |
| Soy |  | NA |
| Bee’s Wax |  | NA |
| EO1 |  |  |
| EO2 |  | Not provided on label. |
| EO3 |  |  |

**Supplemental Table 1 continued: Images of candle types.**

| EO4 |  |  |
| --- | --- | --- |
| EO5 |  |  |
| EO6 |  |  |
| EO7 |  |  |
